# A SARS‐CoV‐2 EG.5 mRNA vaccine induces a broad‐spectrum immune response in mice

**DOI:** 10.1002/mco2.779

**Published:** 2025-01-02

**Authors:** Hongyu Wang, Qinhua Peng, Xinxian Dai, Zhifang Ying, Xiaohong Wu, Xinyu Liu, Hongshan Xu, Jia Li, Leitai Shi, Jingjing Liu, Yunpeng Wang, Danhua Zhao, Yanqiu Huang, Lihong Yang, Ren Yang, Guangzhi Yue, Yue Suo, Qiang Ye, Shouchun Cao, Yuhua Li

**Affiliations:** ^1^ Department of Arboviral Vaccine National Institutes for Food and Drug Control Beijing China; ^2^ Etiology Laboratory，National Vaccine and Serum Institute Beijing China; ^3^ Division of Respiratory Virus Vaccines National Institutes for Food and Drug Control Beijing China

**Keywords:** immunization strategies, immunogenicity, mRNA‐based vaccines, SARS‐CoV‐2 variants

## Abstract

The emerging of emergent SARS‐CoV‐2 subvariants has reduced the protective efficacy of COVID‐19 vaccines. Therefore, novel COVID‐19 vaccines targeting these emergent variants are needed. We designed and prepared CoV072, an mRNA‐based vaccine against SARS‐CoV‐2 Omicron (EG.5) and other emergent SARS‐CoV‐2 subvariants that encodes the EG.5 spike protein. Six‐week‐old female BALB/C mice were used to assess humoral and cellular immune responses and cross‐reactive neutralizing activity against various SARS‐CoV‐2 subvariants. Meanwhile different immunization strategies and doses were performed to detect the immunogenicity of this mRNA vaccine. Our results show that two doses of 5 µg CoV072 or a single dose of 15 µg CoV072 both induced broad‐spectrum cross‐protection ability in mice. Compared with a single dose of 15 µg CoV072, two doses of 5 µg COV072 exhibited higher levels of pseudovirus neutralizing antibody (PNAb) and cross‐reactive IgG responses to multiple variants. Moreover, higher levels of neutralizing antibody (NAb) against live XBB and EG.5 variants were also induced. Th1‐biased cellular immune response was induced in all vaccination groups. The antigen design and immunization strategy of this study have reference significance for the research of the next generation of COVID‐19 vaccine and other vaccines.

## Graphical abstract

Considering that SARS‐CoV‐2 infection cases continue to appear worldwide, and the continuous mutation of the virus has reduced the protective effectiveness of existing vaccines, this study designed a novel vaccine to provide effective protection against SARS‐CoV‐2 variant strains. It will provide reference value for the research and development of the next generation of COVID‐19 vaccine and other related vaccines

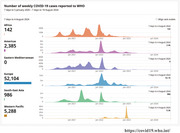

## 1 INTRODUCTION

COVID‐19 has continued to spread globally since end 2019, and the causative virus, SARS‐CoV‐2, is continuously evolving. On November 26 2021, the World Health Organization (WHO) recognized Omicron as a new sublineage of SARS‐CoV‐2 that had spread so rapidly and widely that it had become the predominant endemic subvariant in several countries in only three months.^1^ Omicron sublineages are susceptible to mutations in the spike (S) protein due to epidemiological evolutionary pressures, which lead to an increased number of subvariants and the initiation of widespread competing epidemics.^2^ In late 2021, the XBB sublineage resulting from recombination of BA2.10.1 and BA2.75 was first discovered in India. This sublineage has low pathogenicity but is highly transmissible and infective.^3^, ^4^ In February 2022, the XBB.1.5 subvariant, a descendant of the XBB sublineage, accounted for 49.1% of COVID‐19 cases in the United States^5^ and became the dominant variant worldwide.^6^ As a descendant of the BA.2 sublineage, XBB.1.5 has a mutant S protein that contains the F486P mutation, which increases its affinity for the angiotensin‐converting enzyme 2 receptor, thus increasing infectivity.^5^ Other subvariant of XBB, including XBB.1.9.1, XBB.1.16, and XBB.2.3, are also spreading rapidly worldwide.^7^

EG.5 (Eris) was first reported and classified as a variant under monitoring (VUM) by the WHO on February 17 2023. EG.5 and its subvariants EG.5.1, EG.5.1.1, and EG.5.2 were classified as variants of interest (VOI) on August 9 2023 based on the initial risk assessment of EG.5 by the WHO.^8^ The EG.5 sublineage, a descendant of XBB.1.9.2, spread rapidly and became the dominant sublineage in many countries in North America, Asia, and Europe.^9^, ^10^ Compared to XBB1.5, EG.5 has an additional F456L mutation in the receptor‐binding domain (RBD). This amino acid change has been shown to help EG.5.1 efficiently evade humoral immunity induced by various variants (including the XBB sublineage).^11^, ^12^ Convalescent sera of patients infected with the BQ.1 or XBB sublineage exhibited a 1.3‐ to 2‐fold decrease in neutralizing activity against the EG.5.1 variant compared to that against the XBB.1.5 variant.^13^, ^14^, ^15^, ^16^ In addition, the global prevalence of EG.5 has been steadily increasing. According to the WHO, the percentage of COVID‐19 cases worldwide caused by EG.5 during October 2–8 2023 was 47.0%, which increased to 51.6% only four weeks later (October 30 to November 5 2023).^7^ During this period, the prevalence of EG.5 in China increased from 92.2% to 98.2%.^8^ Although the EG.5 subvariant exhibits an enhanced transmission rate, growth dominance, and immune escape, disease severity did not change and therefore, the public health safety risk of the EG.5 subvariant is low.^8^ The WHO and the Technical Advisory Group on SARS‐CoV‐2 Virus Evolution recommended that member states continue to share information on the growth changes of the EG.5 subvariant and human serum neutralization results.^8^ The Technical Advisory Group on COVID‐19 Vaccine Composition also recommended the continued evaluation of the impact of variants on the efficacy of existing vaccines to inform updates to vaccine composition.^17^

With the emergence of the Omicron variant, global public health strategies and vaccine development face new challenges, as it exhibits stronger immune escape properties and poses a severe test for the protective effect of existing vaccines. Booster immunizations with existing bivalent mRNA vaccines (containing WuHan‐Hu‐1 and BA.4/5) show very limited improvement in the immune responses against Omicron subvariants compared to three doses of monovalent mRNA.^4^, ^18^, ^19^

One study showed that, after three doses of BNT162b2 or mRNA‐1273 vaccine, although the effectiveness of the Delta variant remained at a high level of 88.5%, the effectiveness of the Omicron variant decreased to 66.3%.^20^ Further research data indicated that after two doses of BNT162b2 inoculation, the level of pseudovirus neutralizing antibody against the original strain Wudlamy‐HU‐1 was high, but the level of antibody against Omicron subtype such as BA.1 and Ba.1 variant was significantly decreased.^21^ In addition, after two doses of vaccine, the third dose was given to enhance immunity. Although the booster dose significantly increased the level of neutralizing antibodies, the protection against Omicron variant strains was still limited, and the neutralization capacity against Omicron subtype was still lower than that against the original strain. Two doses of the BNT162b2 vaccine provide only limited protection against symptoms caused by B.1.1.529 infection, with a marked decline in vaccine effectiveness at 20 weeks or more, with greater declines in older and clinically vulnerable populations.^22^

In addition, the protection against currently prevalent variants is less effective than that against previous variants.^18^ Therefore, novel vaccine formulations are needed to combat the constantly changing nature of SARS‐CoV‐2. On June 5 2023, the US Vaccines and Related Biological Products Advisory Committee convened and evaluated the immune protection provided by vaccine candidates by detecting viruses and conducting genomic analysis, antigenic characterization, and human serological testing of existing vaccines. Based on their findings, the committee recommended that the 2023–2024 US COVID‐19 vaccine dosing regimen should include the XBB.1.5 variant.^23^ By testing the neutralizing activity of convalescent sera from patients infected with the XBB.1.5 variant against the XBB.1.5+F456L pseudovirus (mimicking the S protein of EG.5), it was found that the F456L mutation greatly reduced the neutralizing activity in convalescent sera.^18^ Therefore, it is essential to design a novel vaccine containing the F456L mutation to combat the global spread of EG.5 and the possible emergence of new variants.

## 2 RESULTS

### 2.1 mRNA vaccine construction and characteristics

The mRNA vaccine CoV072 uses the S protein of SARS‐CoV‐2 Omicron (EG.5) subvariants as an antigen, which is human codon‐optimized and cloned into a plasmid containing UTRs and poly(A) tails. The mRNA was obtained by in vitro transcription, and then the mRNA transcripts were encapsulated in LNPs. The resulting mRNA‐LNPs had an average particle size of 76.06 nm and a particle dispersion index (PDI) of 0.111, no micron‐sized particles were detected (Figure S1), and the encapsulation efficiency was above 82%. CoV072 mRNA vaccine was directly transfected into HEK293T cells in 24‐well plates at concentrations of 2 µg/well and 4 µg/well, respectively. The untransfected cells were set as negative controls. Use SARS‐CoV‐2 Omicron (B.1.1.529) variant Spike ELISA Kit (cat#: KIT40591C, sino biological), ELISA results showed that CoV072 mRNA vaccine successfully expressed the target protein in vitro (Figure S2).

### 2.2 CoV072 triggers cellular immunity to combat SARS‐CoV‐2 variants of mice

**FIGURE 1:**
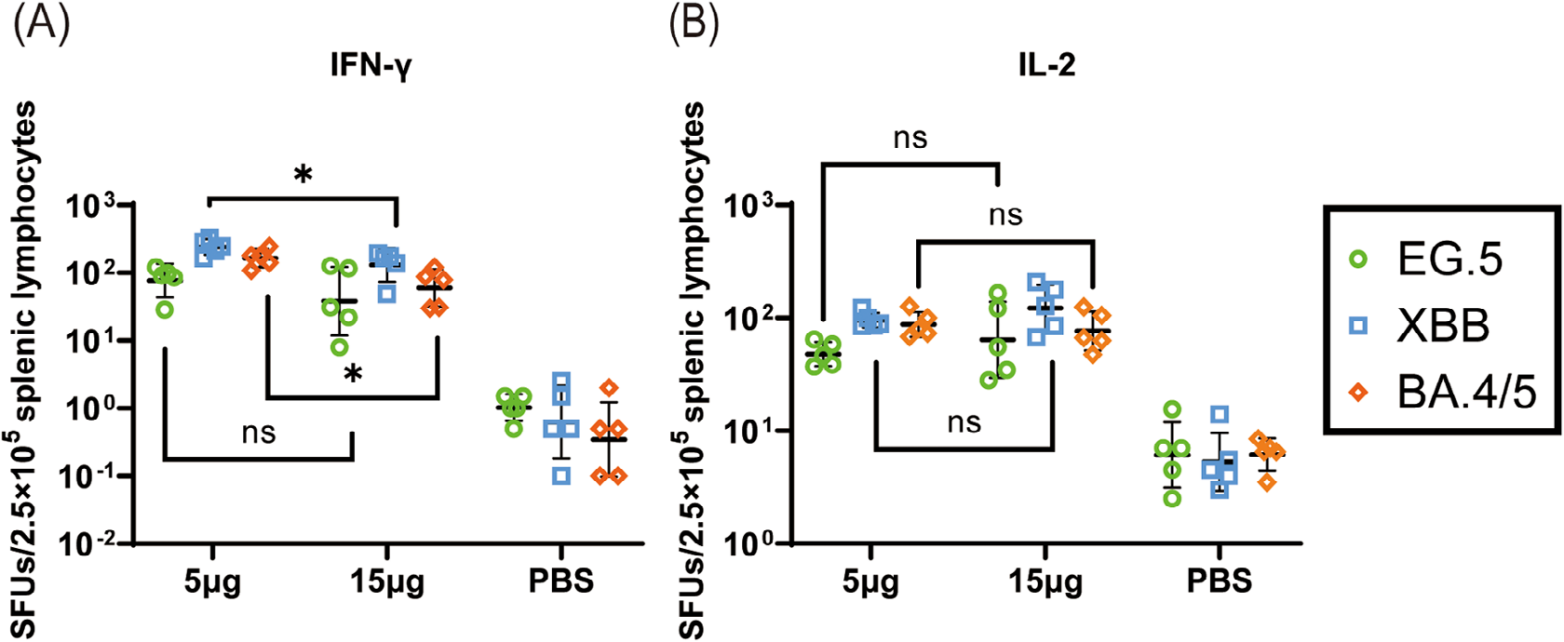
CoV072 induces cellular immunity to combat SARS‐CoV‐2 variants of mice. CoV071‐induced S protein‐specific cellular immune responses were assayed by stimulating mice splenic lymphocytes using S protein peptide libraries from the Omicron BA.4/5, XBB.1.5, and EG.5.1 subvariants followed by ELISpot assays to detect Spike protein‐specific (A) IFN‐γ and (B) IL‐2 secretion in mice splenic lymphocytes. *N* = 5, geometric mean ± geometric standard deviation. *p < 0.05; **p < 0.01; ***p < 0.001; ****p < 0.0001; ns: *p* > 0.05.

Mice were euthanized and splenic lymphocytes were collected 14 days after the primary immunization in the 15 µg single dose group and 35 days after the primary immunization in the 5 µg two doses group. The lymphocytes were stimulated with S protein peptide pools from the Omicron BA.4/5, XBB.1, and EG.5 subvariants for 24 h. After stimulation, ELISpot was used to detect S protein‐specific IFN‐γ and IL‐2 secretion (Figure 1). CoV072 induced a broad‐spectrum cellular immune response in the mice. The 5 µg two doses group had higher levels of IFN‐γ production than the 15 µg single dose group. In the 15 µg single dose group, the geometric mean titers (GMTs) of spot‐forming units SFUs per 2.5 × 10^5^ splenic lymphocytes against the Omicron BA.4/5, XBB.1.5, and EG.5.1 subvariants were 38, 130, and 60, respectively, compared to 76, 240, and 164, respectively, in the 5 µg two doses group, which were 2‐, 1.8‐, and 2.7‐fold higher than those in the 15 µg single dose group, respectively. Both immunization protocols induced similar levels of IL‐2 production. The GMTs of spot‐forming units per 2.5 × 10^5^ splenic lymphocytes against the Omicron BA.4/5, XBB.1.5, and EG.5.1 subvariants were 64, 122, and 76, respectively, for the 15 µg single dose group, and 48, 95, and 87, respectively, for the 5 µg two doses group.

### 2.3 CoV072 induces the Th1‐biased cellular immunity of mice

**FIGURE 2:**
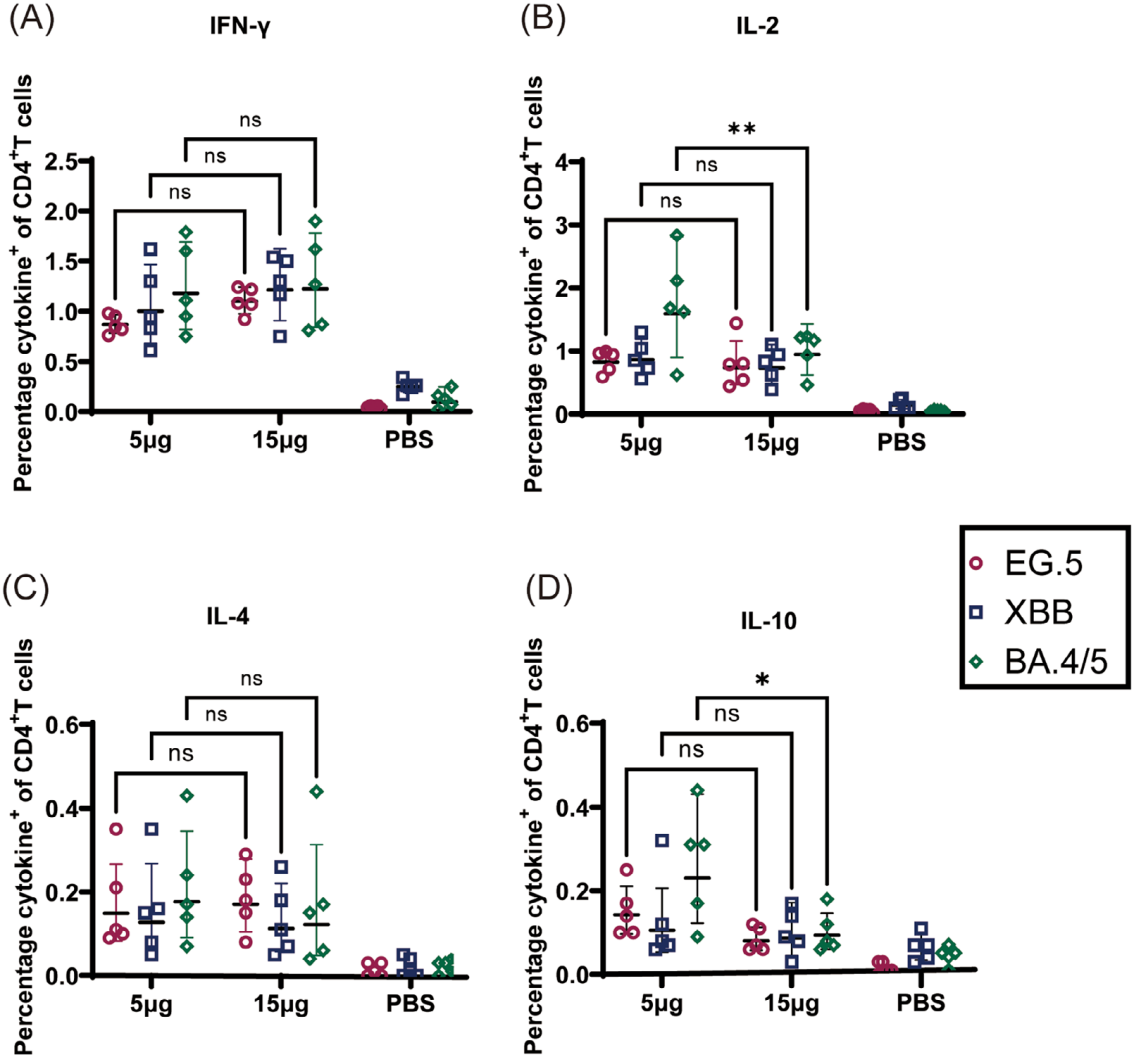
CoV072 induces a Th1‐biased cellular immune response in mice. Intracellular cytokine staining was performed to detect Th1/Th2 bias in the CoV072‐induced cellular immune response. S protein peptide pools from Omicron BA.4/5, XBB.1.5, and EG.5.1 were used to stimulate mouse splenic lymphocytes, and the (A) IFN‐γ, (B) IL‐2, (C) IL‐4, and (D) IL‐10 levels secreted from CD4+ T cells were determined. *N* = 5, geometric mean ± geometric standard deviation. *p < 0.05; **p < 0.01; ***p < 0.001; ****p < 0.0001; ns: *p* > 0.05.

**FIGURE 3:**
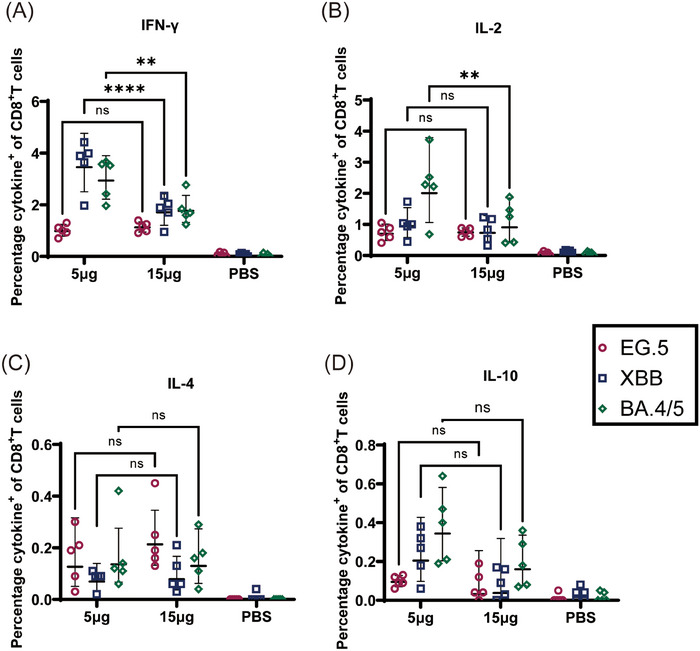
CoV072 induces a Th1‐biased cellular immune response in mice. Intracellular cytokine staining was performed to detect Th1/Th2 bias in the CoV072‐induced cellular immune response. S protein peptide libraries from Omicron BA.4/5, XBB.1.5, and EG.5.1 were used to stimulate mouse splenic lymphocytes, and the (A) IFN‐γ, (B) IL‐2, (C) IL‐4, and (D) IL‐10 levels secreted from CD8+ T cells were determined. *N* = 5, geometric mean ± geometric standard deviation. *p < 0.05; **p < 0.01; ***p < 0.001; ****p < 0.0001; ns: *p* > 0.05.

Next, we assessed the Th1/Th2 bias of the S protein‐specific T‐cell immune response. We harvested mice splenic lymphocytes and stained for them intracellular cytokines after stimulation using S protein peptide pools in Omicron BA.4/5, XBB.1, and EG5.1 subvariants to determine whether CoV072 induced of a Th1‐biased T‐cell immune response against different subvariants in mice. As shown by intracellular cytokine staining (Figures 2 and 3), CD4+ and CD8+ T‐cell proportion that secreted Th1 cytokines (IFN‐γ/IL‐2) was increased in the 5 µg two doses group and 15 µg single dose group compared to the PBS group. However, CD4+ and CD8+ T‐cell proportion that secreted Th2 cytokines (IL‐4/IL‐10) showed no significant difference in two vaccine dose groups compared with PBS group. These findings demonstrated that CoV072 induces a Th1‐biased T‐cell immunity of mice.

### 2.4 CoV072 induces mice to produce high titers of serum IgG antibodies against subvariants

**FIGURE 4:**
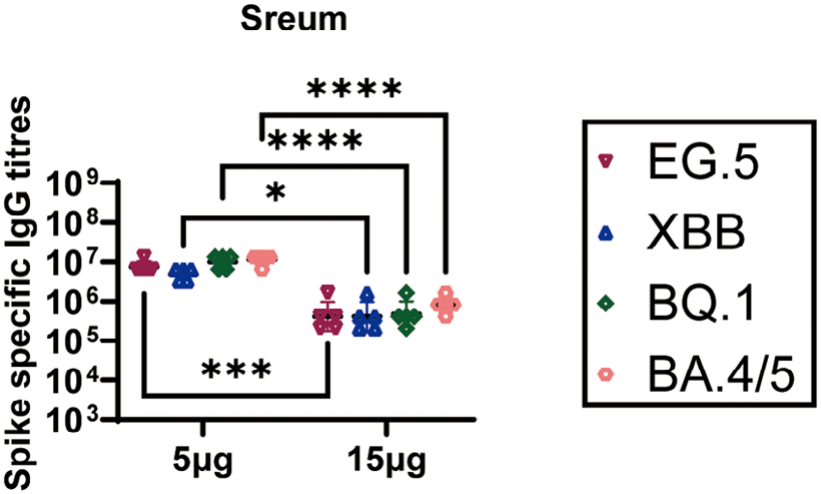
S protein‐specific IgG titers induced by CoV072 in mice. Serum was collected from mice 35 days after the primary immunization in the 5 µg two groups and 14 days after the primary immunization in the 15 µg single group. S protein‐specific IgG antibody titers against Omicron EG.5, Omicron XBB.1, Omicron BQ.1, and Omicron BA.4/5 variant were determined by ELISA. *N* = 5, geometric mean ± geometric standard deviation. *p < 0.05; **p < 0.01; ***p < 0.001; ****p < 0.0001; ns: *p* > 0.05.

ELISA was used to detect S protein‐specific IgG titers in the 5 µg two doses group and 15 µg single dose group to assess the level of humoral immune responses induced by CoV072 in mice. Omicron BA.4, BQ.1, XBB.1, and EG.5.1 S protein‐specific IgG titers are shown in Figure 4. The 5 µg two doses group exhibited higher levels of cross‐reactive IgG responses than the 15 µg single dose group, with GMTs of 11,410,480, 9,933,400, 4,966,700, and 7,528,110, respectively, against the above four subvariants. The highest S protein‐specific IgG titer was observed for the Omicron BA.4 variant and it was 1.1‐, 2.3‐, and 1.5‐fold higher than those for the other three subvariants. In the 15 µg single dose group, the GMTs were 819,200, 470,507, 409,600, and 409,600 for the above four variants, respectively, which were 13.9‐, 21.1‐, 12.1‐, and 18.4‐fold lower, respectively, than those in the 5 µg two doses group.

### 2.5 CoV072 induces mice to produce broad‐spectrum neutralizing antibodies against multiple subvariants

**FIGURE 5:**
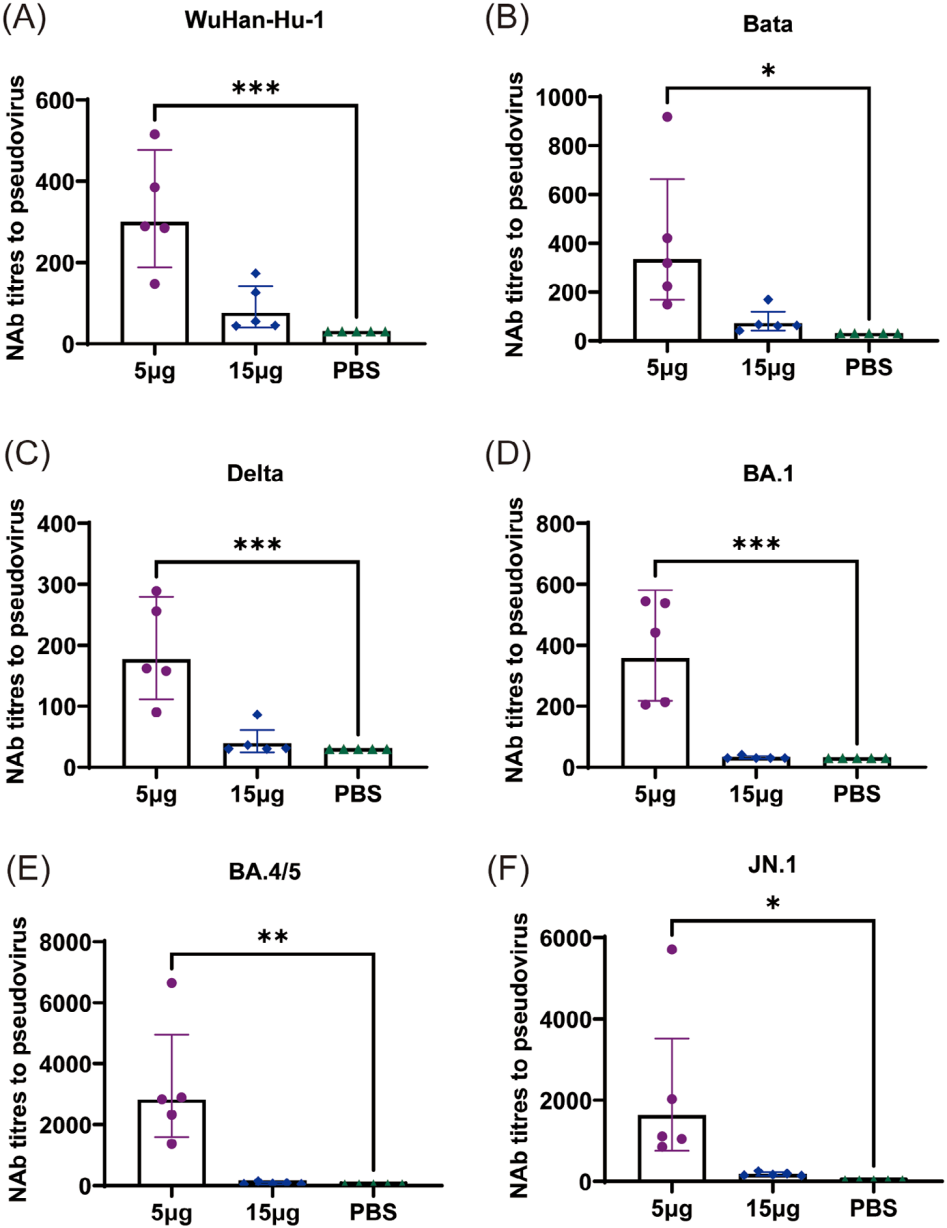
Neutralizing antibodies levels to resist SARS‐CoV‐2 pseudovirus. NAb titer was determined 35 days after primary immunization in the 5 µg two groups and 14 days after the primary immunization in the 15 µg single group. Serum NAb titers against PNAb titers of (A) WuHan‐Hu‐1, (B) Beta, (C) Delta, (D) Omicron BA.1, (E) Omicron BA.4/5, and (F) Omicron subvariants at IC50. *n* = 5, geometric mean ± geometric standard deviation. *p < 0.05; **p < 0.01; ***p < 0.001; ****p < 0.0001; ns: *p* > 0.05.

VSV pseudoviruses were used to determine neutralizing antibody titers against the WuHan‐Hu‐1, Beta, Delta, Omicron BA.1, Omicron BA.4/5, and JN.1 variants in mice sera (Figure 5). The 5 µg two doses group exhibited superior induction of pseudovirus neutralizing antibodies (PNAb) against these six variants to 15 µg single‐dose and PBS groups. GMTs of PNAb production were 299, 333, 176, 356, 2809, and 1627, respectively, in the 5 µg two doses group and 75, 71, 39, 32, 73, and 179, respectively, in the 15 µg single dose group, which were 4.0‐, 4.7‐, 4.5‐,11.1‐, 38.5‐, and 9.1‐fold lower, respectively, than those in the 5 µg two doses group. Based on these data, a low‐dose mRNA vaccine at two doses induces higher humoral immunity levels than a single dose.

**FIGURE 6:**
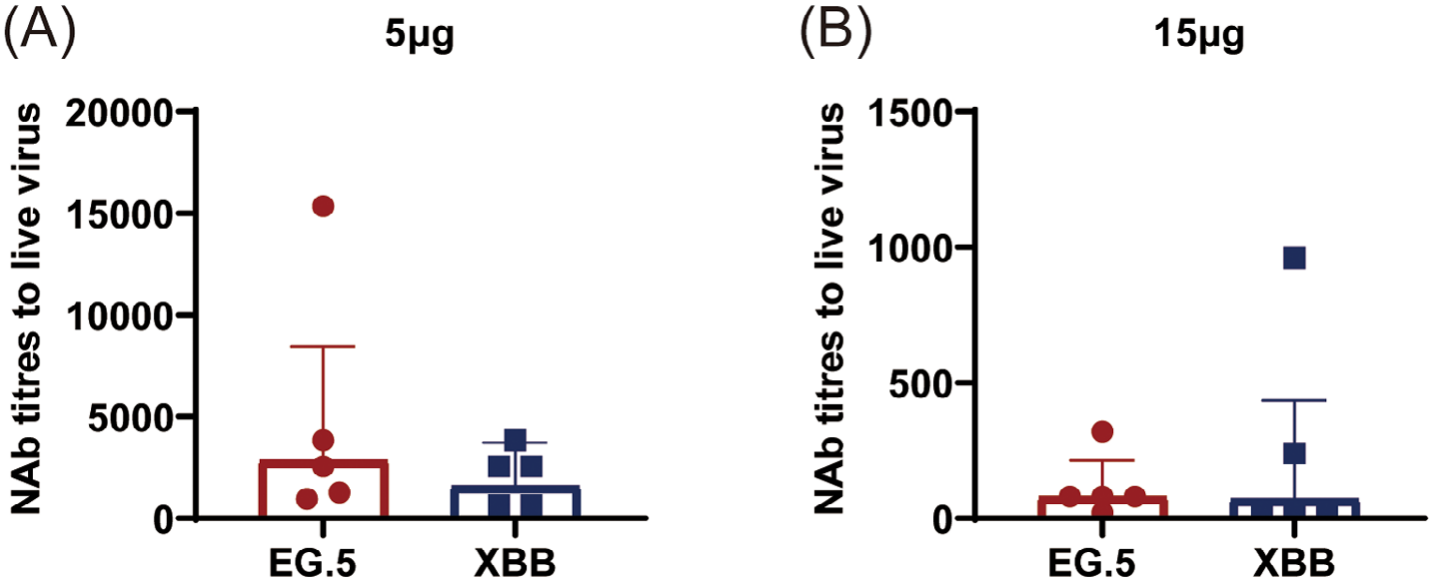
Neutralizing antibodies levels to resist SARS‐CoV‐2 live virus. NAb titer was determined on day 35 following primary immunization in the 5 µg (A) two doses group and 14 days after the primary immunization in the 15 µg (B) single dose group. Serum NAb titers EG.5 and XBB.1 subvariants at IC50. *n* = 5, geometric mean ± geometric standard deviation. *p < 0.05; **p < 0.01; ***p < 0.001; ****p < 0.0001; ns: *p* > 0.05.

To evaluate the titers of neutralizing antibodies (NAb) induced by CoV072 in mice against live XBB.1 and EG.5.1 subvariants, the serum NAb content was determined using a virus‐specific MN assay. The GMTs of NAb to resist XBB.1 and EG.5.1 subvariants were 1595 and 2842, respectively, in the 5 µg two doses group, which were significantly higher (22.5‐ and 35.5‐fold) than those in the 15 µg single dose group (71 and 80, respectively) (Figure 6). Thus, 5 µg mRNA‐based vaccine at two doses produces the better humoral immunity than a single dose of 15 µg.

## 3 DISCUSSION

SARS‐CoV‐2 keeps on evolution, which continues to have an important impact on national public health systems, with the XBB and EG.5 subvariants being the most important contributors to the recent surge in COVID‐19 cases.^17^ In particular, the EG.5 sublineage exhibited significant challenges to the existing SARS‐CoV‐2 vaccine efficacy. The bivalent mRNA vaccine (consisting of Wuhan‐Hu‐1 and BA.4/5) was authorized by the US Food and Drug Administration in emergency in response to persistent mutations in the virus, but NAb levels triggered in the body by this vaccine was substantially lower than that achieved with the original D614G variant. In addition, convalescent sera of patients infected with BA.4/5 or XBB.1.5 had a substantially reduced ability to neutralize the EG.5 sublineage.^24^ Therefore, developing novel mRNA vaccines to combat emergent SARS‐CoV‐2 variants and increasing immune response level against them are major priorities. In the present study, we developed the CoV072 vaccine, encoding the S protein of the EG.5 sublineage. The greatest broad cross‐reactive IgG and NAb response and Th1‐bias cellular immune response levels were achieved with 5 µg two doses group as compared to 15 µg single dose group. Thus, CoV072 induces a broad protective effect in response to de novo variants and can be considered a promising novel COVID‐19 vaccine candidate.

CoV072 induced a high‐level humoral immunity to resist XBB.1 and EG.5.1. However, sera from patients infected with the XBB sublineage are unexpected in neutralizing the EG.5.1 subvariant.^13^, ^14^, ^15^, ^16^, ^17^ This may be related to the mutation sites in EG.5.1. The RBD region of EG.5.1 contains an additional F456L mutation compared to that of XBB.1.^25^ Kaku et al. determined the 50% neutralizing titers (NT50) of pseudoviruses XBB.1.5, XBB.1.9.2, and EG.5.1 using convalescent sera of patients infected with the XBB sublineage and found that the NT_50_ against the EG.5.1 pseudovirus was decreased by 1.4‐fold. In addition, the NT_50_ against the modified XBB.1.5/1.9.2+F456L pseudovirus decreased by 1.9‐time in comparison with XBB.1.5 and XBB.1.9.2.^13^ This reinforces the importance of F456L as an important site in the design of novel vaccines for generating an immune response against EG.5.1. According to the live virus neutralizing antibody assay, GMTs of NAb for resisting XBB.1 and EG.5.1 were 1595 and 2842 respectively; the NAb of EG.5.1 was increased due to the introduction of F456L.

On September 29 2023, a variant carrying the L455S mutation in the S protein was named JN.1 by the PANGO Network.^26^ JN.1, the BA.2.86 descendant, possesses only one more mutation site (i.e., L455S) within S protein compared to BA.2.86.^27^ In contrast, BA.2.86, the BA.2 descendant,^28^ carries several mutations within S protein compared to EG.5.1. However, it is also a sublineage of Omicron, and both contain the key mutation sites K417N, S477N, E484K, N501Y, and P681H.^24^ In a recent study characterizing the JN.1 variant, Kuka et al.^29^ reported that the presence of L455S did not contribute to the difference in antigenicity between JN.1 and BA.2.86. Furthermore, serum samples in XBB.1.5 or EG.5.1 infected patients had a 3.8‐fold decreased ability to neutralize JN.1 compared to sera from patients infected with BA.2.86. Finally, the presence of L455S results in JN.1 exhibiting high immune escape against sera from XBB.1.5 vaccination. Recently, according to Wang et al., booster immunization with a monovalent XBB.1.5 vaccine led to high cross‐protection from JN.1 despite the large mutational differences between XBB.1.5 and JN.1.^30^ Therefore, the use of a COVID‐19 vaccine with a similar mutation to XBB.1.5 may help prevent severe COVID‐19 patients, consistent with our results. In the 5 µg two doses group, mice sera retained high neutralization activity against JN.1 pseudovirus, with a GMTs of 1627.

Our results show that two doses of the 5 µg CoV072 mRNA vaccine produced a higher humoral immune response than a single dose of the 15 µg CoV072. From an immunological standpoint, this could be attributed to the enhanced memory effect of the immune system following multiple vaccinations. The prime immunity initiates the immune response, while the booster immunity helps to consolidate this response, Leads to enhanced antibody levels and T‐cell response. Within a specific dose range, increasing the dose enhances the immune response; however, once the dose surpasses a certain threshold, continuing to increase the dose does not significantly enhance the immune response. In fact, high doses of vaccination may lead to immune tolerance. A study by Moderna showed that the immune effect of a dose of 25 µg was comparable to that produced by natural infection with SARS‐CoV‐2, indicating the effectiveness of lower doses.^31^ In addition, both Pfizer /BioNTech and Moderna mRNA vaccines have been tested in clinical trials following a two‐dose regimen, and the results show that two doses of the vaccine are highly protective. It is clear from existing studies and practical applications that multi‐dose mRNA vaccines, especially at reasonable doses, can provide better immune effects. This is mainly because multiple vaccinations enhance immune memory and may reduce side effects, ensuring that a wider population can complete the immunization program and receive ongoing protection.

Our results show that the CoV072 vaccine induces broad‐spectrum cross‐protection ability in mice, but neutralizing antibody levels against Wuhan‐HU‐1, Beta, and Delta variants are significantly lower than those against Omicron variants. Thimmiraju et al.^32^ assayed neutralizing antibodies in sera of mice administered with XBB.1.5 RBD and found lower antibody titers to resist WuHan‐Hu‐1, Beta, and Delta variants. Such results were closely associated with the Spike protein structures of the variants. For example, Spike protein in EG.5.1 has 42 mutations in comparison with the WuHan‐Hu‐1 strain.^23^ In a study administering a mRNA vaccine for the XBB.1.5 variant as a booster for people receiving several vaccine doses, sera from these individuals still contained high NAb titers for WuHan‐Hu‐1+D614G variant.^33^ This suggests that CoV072 may be a good booster vaccine candidate. This is consistent with the current strategy advocated in China of vaccines prepared with the current various to protect against the current COVID‐19 epidemic.

CoV072 induces broad‐spectrum cellular and humoral immunity to resist various variants. It is efficacious for the currently circulating variants, and may provide good protection against future variants of this lineage. Our findings conform to current strategy advocated in China of immunizing high‐risk populations with COVID‐19 vaccines that contain XBB variant. COVID‐19 vaccines that contain XBB variant approved for use in China provide better protection against the XBB and JN sublineages.^34^ Fudan University recently reported that a COVID‐19 vaccine containing the XBB variant approved in China provides better protection against the XBB and JN sublineages, which is also consistent with our findings.^35^

Although this study has achieved positive results under existing experimental conditions, there are still some limitations. Due to the limitations of experimental materials and experimental environment, this study lacks experiments on the protective efficacy of animal models. The existing experimental results show that the serum IgG and neutralizing antibody contents and cytokine levels of mice injected with CoV072 vaccine are significantly increased. mRNA vaccines induce mice to produce high levels of neutralizing antibodies that effectively recognize and neutralize the virus, block its entry into cells and reduce viral load. Moreover, CD4+ and CD8+ T cells produced in mice were able to secrete a variety of cytokines, such as IFN‐γ and TNF‐α, when stimulated by S proteins, showing a significant Th1‐biased immune response. This Th1 bias helps activate macrophages and promotes the development of cytotoxic T cells that directly attack virus‐infected cells.^36^ Therefore, humoral immunity and cellular immunity together constitute an effective defense mechanism against SARS‐CoV‐2. Based on this, we speculate that in the animal challenge experiment, vaccinated animals will have significantly lower viral loads in their respiratory tract and blood after the virus attack than unvaccinated controls, as well as less weight loss and faster recovery after infection. Kackos et al.^37^ confirmed that vaccination with GLB‐COV2‐043 induces a strong antigen‐specific binding antibody and virus‐neutralizing antibody response in mice. Similarly, in hamster models, infection with the Omicron BA.1 variant resulted in reduced viral load, reduced pathological changes in the lungs, and effective protection against weight loss after vaccination. These findings further support our hypothesis.

In conclusion, SARS‐CoV‐2 subvariant EG.5 mRNA vaccine CoV072 induces broad‐spectrum cellular and humoral immunity to resist various SARS‐CoV‐2 variants. In addition, we found Two low doses of immunization are more effective than a single high dose, which is particularly important in the development of mRNA vaccines. Specifically, vaccine dose may be reduced for mitigating adverse effects, while increasing its efficacy. Our findings lay the certain foundation for developing novel vaccines against SARS‐CoV‐2 variants and other human vaccines and are of major significance for controlling the COVID‐19 epidemic.

## 4 MATERIALS AND METHODS

### 4.1 mRNA vaccine preparation

The CoV702 encodes a Spike protein in the SARS‐CoV‐2 Omicron (EG.5) subvariants. For enhancing the encoded antigen level and release, a natural signal peptide of the S protein was replaced with that of a human tissue‐type plasminogen activator (tPA). A poly‐A tail and untranslated regions were flanked in the coding region for improving mRNA translation efficiency. mRNA was synthesized in vitro using a linearized DNA plasmid to be the template. A cap analogue was added to the mRNA to obtain mRNA with the Cap‐1 structure. DNA template was eliminated and reaction was stopped through adding DNase I in the reaction system. After purification with magnetic RNA purification beads (cat#: N412‐02, Vazyme) from in vitro transcription reaction system, mRNA was eluted with nuclease‐free water before freezing at –80°C. Lipid nanoparticles (LNP) were later adopted to assemble the purified mRNA for forming mRNA‐LNP complexes. An LNP mix was obtained through dissolving ionizable lipids (cat#: 2089251‐47‐6, Sinopeg), polyethylene glycol 2000 (cat#: 160743‐62‐4, Sinopeg), 1,2‐distearoyl‐sn‐glycero‐3‐phosphocholine(DSPC) (cat#: 816‐94‐4, Sinopeg), and cholesterol (cat#: 57885, AVT) in anhydrous ethanol at a molar ratio of 50:1.5:10:38.5. mRNA‐LNP complexes were obtained through rapidly blending LNP mixture with purified mRNA under specific volumetric ratios.

### 4.2 Cells and animals

**FIGURE 7:**
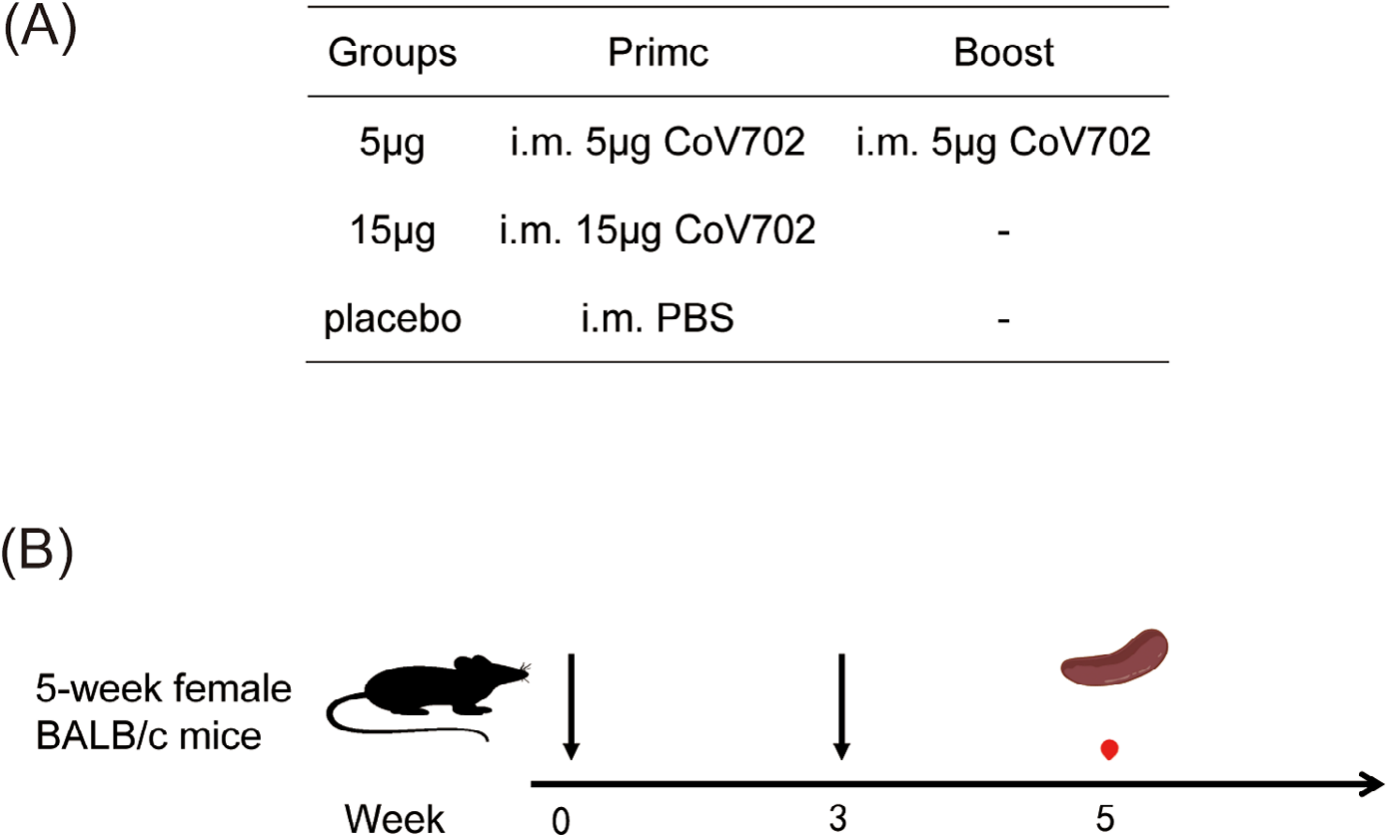
Mice immunization regimens. Note: Prime: prime immunization; Boost: booster immunization; i.m.: intramuscular injection; ‐: no booster immunization; immunization; blood sample collection; separation of lymphocytes from the spleen.

We purchased Vero cells in the American Type Culture Collection and preserved them in National Institutes for Food and Drug Control (NIFDC). Cells were cultivated within DMEM that contained 10% fetal bovine serum as well as 1% antibiotics under 5% CO_2_ and 37°C conditions. Animal experimental protocols gained approval from Institutional Animal Care and Use Committee of NIFDC and conducted following the committee guidelines. Six‐week‐old specific pathogen‐free female BALB/c mice were provided and housed by the Chinese NIFDC. Mice were randomly assigned to a 5 µg, a 15 µg, or a placebo group (*N* = 5 mice/group) (Figure 7). In the 5 µg two doses group, the primary immunization was administered on day 0 with a dose of 5 µg per mice. The booster immunization was administered on day 21 after primary immunization (day 0). Serum was collected 35 days following primary immunization. Thereafter, mouse sacrifice was implemented to collect splenic lymphocytes in subsequent analysis. In the 15 µg single dose group, the primary immunization at 15 µg per mouse was injected after 21 days, and serum was collected on day 14 following primary immunization. In placebo group, PBS was added for mouse inoculation. All mice were euthanized in accordance with relevant guidelines.

### 4.3 Analysis of the cellular immune response using enzyme‐linked immunosorbent spot (ELISpot) assays

After the mice were euthanized and soaked with 75% ethanol for 1 min, splenic tissues were harvested into the 10‐cm cell‐culture dish. Four to five milliliters of mice lymphocyte isolate (cat#: 7211011, Dakewe) was taken, and the splenic tissues were grinded using the 2‐mL syringe plunger, followed by filtration with the 40‐µm filter. Thereafter, we added the spleen cell suspension into the 15‐mL centrifuge tube at once, introduced 1 mL RPMI‐1640 medium (cat#: 42401042, Gibco), and centrifuged the mixture at 800 × *g* at 4°C for a 30‐min duration. Afterward, the solution within centrifuge tube was separated in 4 layers, including the RPMI‐1640 medium, lymphocyte, separation medium layers, and the layer containing red blood cells as well as cell debris, from the top down. After aspiration, we added the lymphocyte layer to 10 mL RPMI‐1640 medium, centrifuged the mixture for a 10‐min period at 250 × *g* and 4°C to harvest lymphocytes, and suspended cells within serum‐free medium. Mouse IFN‐γ (cat#: 3511–4hpw‐2, Mabtech) and IL‐2 ELISpot plus kits (cat#: 3441–4hpw‐2, Mabtech) were then adopted for detecting interferon gamma (IFN‐γ)‐ and interleukin (IL)‐2‐positive cells. 96‐Well PVDF plates were rinsed by PBS (200 µL) twice, prior to 2‐h blocking using RPMI‐1640 medium that contained 10% fetal bovine serum under 25°C. We later added the separated lymphocytes (2.5 × 10^5^ cells) into each well and treated them with 1 µg/mL of S protein peptide pools from the EG.5 (GenScript, Nanjing, China), XBB.1.5 (cat#: DD9123, Vazyme), and BA.4/5 (cat#: DD 9125, Vazyme) subvariants for 24 h under 37°C. Lymphocytes were later subjected to 2‐h incubation using anti‐mouse IFN‐γ or anti‐mouse IL‐2 antibody under ambient temperature and then 1‐h incubation using streptavidin‐horseradish peroxidase (1:1000). Cells were later washed before 5‐min incubation using 3,5,‐tetramethylbenzidine (TMB, 100 µL) substrate solution until visible spots appeared. Employ the ImmunoSpot S6 Universal spot counter for imaging and counting of IFN‐γ‐secreting cell spots.

### 4.4 Intracellular staining for cytokine

S protein peptide pools (2 µg/mL) from the EG.5, XBB.1.5, or BA.4/5 subvariants were used to stimulate isolated splenic lymphocytes at 37°C for 8 h. Next, they were stained with a mixture of PE hamster anti‐mouse CD3e antibody (cat#: 553063, BD), BV510 rat anti‐mouse CD4 antibody (cat#: 563106, BD), FITC rat anti‐mouse CD8a antibody (cat#: 553030, BD), as well as Fixable Viability Stain 780 (cat#: 565388, BD) to exclude dead cells. Cells were subsequently washed by PBS twice prior to permeabilization using Cytofix/Cytoperm (cat#: 555028, BD), washing by Perm/Wash buffer and staining using PE‐Cy™7‐conjugated rat anti‐mouse IFN‐γ (cat#: 557649, BD) or BV605 rat anti‐mouse IL‐2 (cat#: 563911, BD). After washing by PBS and Perm/Wash buffer, cells were subjected to resuspension within PBS and counting by flow cytometry. We obtained 50,000 or more cells in every sample. FlowJo software was adopted for data analysis. CD4+ and CD8+ T cells were gated by single cells (FSC‐A vs. FSC‐H), live CD3+ T cells (CD3+ vs. LD780–), and lymphocytes (FSC‐A vs. SSC‐A). Detection data were represented by cytokine‐positive CD4+ or CD8+ T‐cell percentage.

### 4.5 Analysis on S protein‐specific IgG titers using an enzyme‐linked immunosorbent assay (ELISA)

ELISA was conducted to analyze the spike protein‐specific antibody titers in serum. S protein peptide pools from the Omicron EG.5.1 (cat#: CG284, Vazyme), Omicron XBB.1 (cat#: CG275, Vazyme), Omicron BQ.1 (cat#: CG273, Vazyme), and Omicron BA.4 (cat#: CG246, Vazyme) subvariants (0.2 µg/well) were added to coat the 96‐well plates overnight. After PBST (PBS + 0.05% Tween‐20) washing, PBST that contained 1% bovine serum albumin (BSA) was introduced to block all wells for a 1‐h period under 37°C. After washing by PBST for six times, serial diluted serum was introduced into each well for 1‐h incubation under 37°C. After washing by PBST six times again, the plate was then probed with horseradish peroxidase‐conjugated goat anti‐mouse IgG (1:25,000) for a 1‐h duration under 37°C. Following ringing, the absorbances were detected at 450 and 630 nm with 3,5‐tetramethylbenzidine being a substrate. Serum IgG titer was determined by GraphPad Prism v.9 software. Serum antibody titer was defined as the greatest dilution whose absorbance increased by 2.1‐folds relative to negative control.

### 4.6 Vesicular stomatitis virus (VSV) pseudovirus neutralization assays

Mice serum was inactivated for a 30‐min period within the water bath at 56°C. Serial dilutions of serum were then mixed with VSV‐based SARS‐CoV‐2 pseudoviruses expressing luciferase, including WuHan‐Hu‐1, Beta, Delta, Omicron BA.1, Omicron BA.4/5, and JN.1 pseudoviruses, followed by 1‐h incubation under 37°C. The addition of Vero cells (2 × 10^5^) was then completed for 24‐h incubation under 37°C in the presence of 5% CO2. Relative luciferase activities were assayed using the luciferase assay system. We included both cell and virus controls in the same plate. After calculating the neutralization percentage, Reed‐Muench approach was employed to determine sample's half‐maximal effective concentration (EC50).

### 4.7 Live SARS‐CoV‐2 neutralization assays

The neutralizing ability of mice serum was analyzed with the micro‐neutralization (MN) assay. To be specific, heat‐inactivated serum was first diluted at twofolds before 2‐h exposure to XBB.1 and EG.5 variants at 100 culture infectious dose (CCID50) under 37°C. The addition of Vero E6 cells (1.8 × 10^5^) was conducted for 72‐h incubation under 37°C. MN antibody titer was determined by Spearman–Karber approach for assessing serum dilution necessary to inhibit 50% cytopathic activity, with MN antibody titer ≥4 being deemed as positive. The virus and MN assays were carried out at the biosafety level‐3 facility in the NIFDC.

### 4.8 Statistical analysis

GraphPad Prism v9.5.0 software was employed for all plotting and statistical tests. Data are shown as the geometric mean ± geometric standard deviation. Differences among multiple groups were compared by two‐way ANOVA. *p < 0.05; **p < 0.01; ***p < 0.001; ****p < 0.0001; ns, not significant.

## AUTHOR CONTRIBUTIONS

Yuhua Li, Shouchun Cao, and Qiang Ye provided the research concepts and designed the experiments. Hongyu Wang and Qinhua Peng prepared vaccine and performed animal immunization, ELISpot, flow cytometry, ELISA, and pseudovirus neutralization assays. Xinxian Dai and Zhifang Ying performed MN assays. Xiaohong Wu contributed to the process of data collection. Hongyu Wang drafted the manuscript. Yuhua Li and Qinhua Peng revised and edited the manuscript. All authors have read and approved the final manuscript.

## CONFLICT OF INTEREST STATEMENT

All authors declare no conflict of interest directly related to this work.

## ETHICS STATEMENT

The animal study was reviewed and approved by the Institutional Animal Care and Use Committee of the National Institutes for Food and Drug Control is affiliated with the National Institute for Food and Drug Control. The animal approval number is 019(A)062.

## Supporting information



Supporting information

## Data Availability

All data are available from the corresponding authors upon request.
