# Supplementary material for: A SARS‐CoV‐2 EG.5 mRNA vaccine induces a broad‐spectrum immune response in mice
Source: MedComm (2020). 2025 Jan 2;6(1):e779. doi: 10.1002/mco2.779 (PMC11695206; doi:10.1002/mco2.779)
Supplement: Supplementary file 1 — Supporting information [file MCO2-6-e779-s001.docx]

# *A SARS-CoV-2 EG.5 mRNA vaccine induces a broad-spectrum immune response in mice*


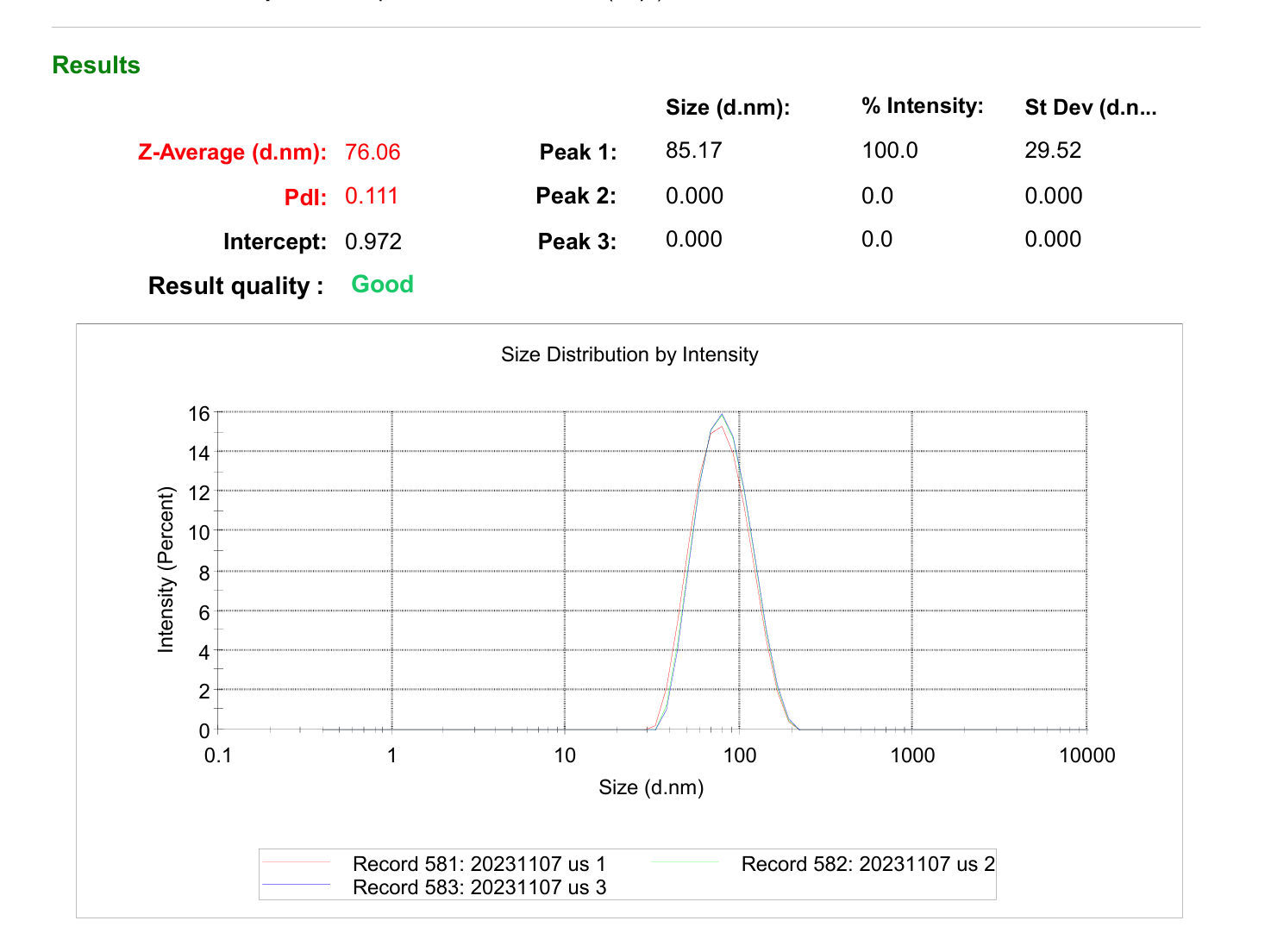


**Figure S1.** Particle size distributions of CoV072


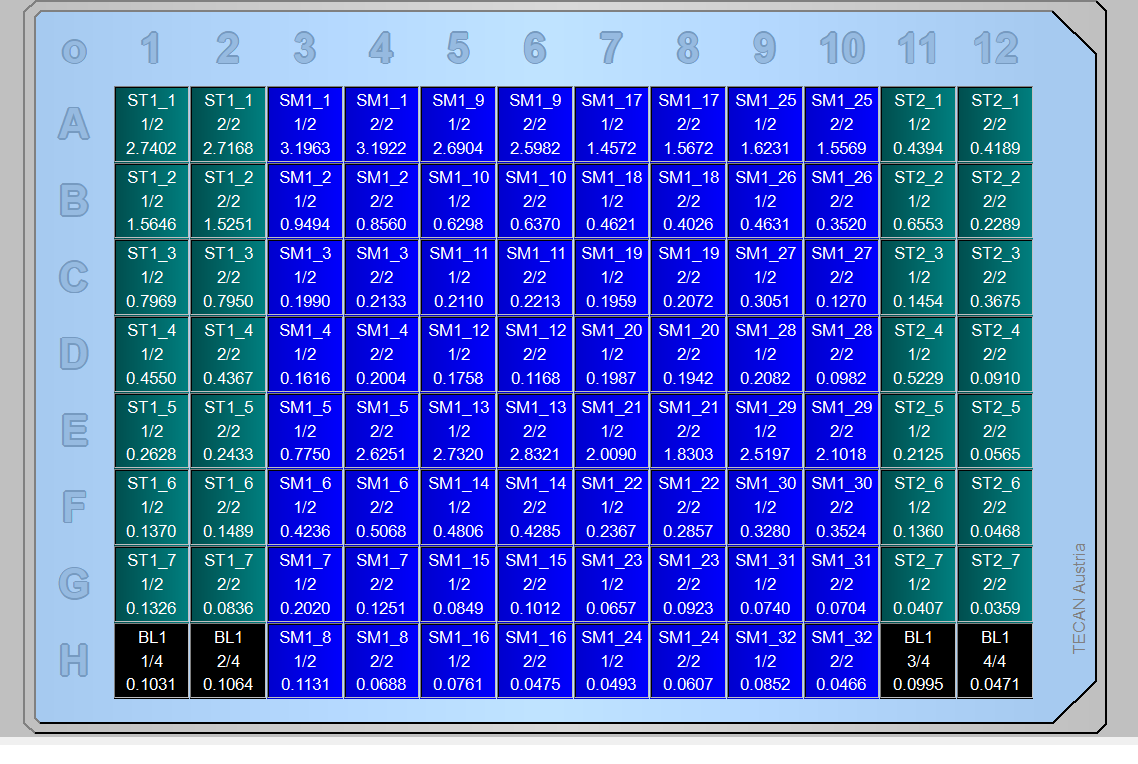


**Figure S2.** EG.5 Spike protein expression in HEK293T cells transfected with CoV072. ST1 is the standard dilution curve of standard substance in the kit, and ST2 is the standard dilution curve of Vazyme's EG.5 S protein.
